# Automatic Irrigation Scheduling on a Hedgerow Olive Orchard Using an Algorithm of Water Balance Readjusted with Soil Moisture Sensors

**DOI:** 10.3390/s20092526

**Published:** 2020-04-29

**Authors:** Sandra Millán, Carlos Campillo, Jaume Casadesús, Juan Manuel Pérez-Rodríguez, Maria Henar Prieto

**Affiliations:** 1 Centre for Scientific and Technological Research of Extremadura (CICYTEX), Department of Horticulture, Finca La Orden, Regional Government of Extremadura, Highway A-V, Km 372, 06187 Guadajira, Badajoz, Spain; carlos.campillo@juntaex.es (C.C.); juanmanuel.perezr@juntaex.es (J.M.P.-R.); henar.prieto@juntaex.es (M.H.P.); 2 Program of Efficient Use of Water in Agriculture, Institute of Agrifood Research and Technology (IRTA), Parc de Gardeny (PCiTAL), Fruitcentre, 25003 Lleida, Spain; jaume.casadesus@irta.cat

**Keywords:** apparent electrical conductivity, NDVI, water use efficiency, precision irrigation, capacitive sensors

## Abstract

Recent technological advances have made possible automated irrigation scheduling using decision-support tools. These tools help farmers to make better decisions in the management of their irrigation system, thus increasing yields while preserving water resources. The aim of this study is to evaluate in a commercial plot an automated irrigation system combined with remote-sensing techniques and soil mapping that allows the establishment of regulated deficit irrigation (RDI) strategies. The study was carried out over 3 years (2015–2017) in a commercial hedgerow olive orchard of the variety ‘Arbequina’ located in Alvarado (Extremadura, Spain). An apparent electrical conductivity (ECa) map and a normalized difference vegetation index (NDVI) map were generated to characterize the spatial variability of the plot and classify the zones in homogeneous areas. Then, reference points were selected to monitor the different irrigation sectors. In 2015, the plot was irrigated according to the farmer’s technical criteria throughout the plot. In 2016 and 2017, two different areas of the plot were irrigated applying an RDI strategy, one under expert supervision and the other automatically. The results show that in a heterogeneous plot the use of new technologies can be useful to establish the ideal location for an automatic irrigation system. Furthermore, automatic irrigation scheduling made it possible to establish an RDI strategy recommended by an expert, resulting in the homogenization of production throughout the plot without the need for human intervention.

## 1. Introduction

The olive (*Olea europaea* L.) tree has traditionally been cultivated in low-density orchards under rainfed conditions due to the drought tolerance capacity of this evergreen species [1]. However, growth and yield may be affected by the decrease in the photosynthetic rate of olive cultivars under rainfed conditions [2]. Since the 1970s there has been a steady increase in the land area occupied by irrigated olive orchards, which was accelerated by the appearance of super-high density plantations (SHD, 1500–2000 trees/ha) in the 1990s. The main advantage of highly mechanized SHD systems is the reduction in labor costs during pruning and harvesting. However, such systems require specific agronomic techniques and are very costly to set up [3]. Irrigation is very important in SHD olive groves to ensure high productivity, as the trees have a limited root volume, a high leaf area index and, in consequence, high water demands [4,5]. However, the application of excessive amounts of water can lead to uncontrolled vigor, the need for severe pruning to adapt the hedgerow to the operation of the mechanical harvester, and low lighting in the fruiting areas, producing an imbalance between growth and productivity [6].

Furthermore, water is a scarce resource [7]. According to the Food and Agriculture Organization (FAO) of the United Nations, agriculture is responsible for over 70% of worldwide water consumption, and it is estimated that the amounts used for irrigation will rise by 14% in the next 10 years [8]. Therefore, to cope with water scarcity and to improve the profitability of SHD olive groves, water savings irrigation strategies must be used in order to control hedgerow vigor, as canopies with greater productive efficiency are key to increasing the viability of these systems [9]. RDI is a management strategy that imposes water deficits in phenological stages less sensitive to drought in order to restrain vegetative growth while not negatively impacting yield and fruit quality [10,11]. The phenological stage that is least sensitive to water deficit in the olive tree is the period from pit hardening to veraison [12,13].

Irrigation scheduling requires decision-making with respect to when and how much irrigation should be applied according to crop type, crop development and environmental conditions. The soil water balance (WB) method is widely used to determine the irrigation needs of a crop, where the water inputs to the soil-plant system must be balanced with the expected outputs. The most important component of the WB is the crop evapotranspiration (ETc) value, which is the crop water need that considers both evaporation from the soil and transpiration from the plants. The ETc is estimated as the product of the evapotranspiration of a reference crop (ETo) and a crop coefficient, Kc, in the form ETc = ETo × Kc [14]. In this relationship, ETo represents the demand imposed by the meteorological conditions while Kc integrates the physical and biophysical differences between the reference crop and the crop which is to be estimated for evapotranspiration [14]. Irrigation scheduling based on WB presents the advantage of anticipating crop water requirements at certain times during the growing season and the possibility of planning irrigation accordingly [15]. However, this method presents the disadvantage that predicted ETc values could be inaccurate because of changes in annual weather patterns and differences in the production practices for which the Kc was developed [16]. An alternative to the WB-based method is to use soil moisture sensors to help plan irrigation scheduling. This method considers the soil as a water reserve for plant growth, and the idea is to ensure the reserve always has a sufficient amount of water available to the plant. Irrigation control is based on the monitoring and measurements of soil water content or water potential. Various types of these sensors have been used to determine soil water content [17]. In addition to the problem of having to weigh up the pros and cons of the different sensor types, the appropriate placement of sensors to accurately reflect the conditions experienced by the plant can be challenging [18]. Consideration needs to be given to the fact that soil water content patterns in the root zone are dynamic and influenced by soil hydraulic properties, spatial heterogeneity, crop characteristics and the irrigation system, among others. In drip irrigation, the local application of irrigation water results in even higher spatial variability in the soil water content patterns formed under the emitters [19]. In general, given the benefits and drawbacks of the WB-based and soil water content monitoring methods, combining both approaches seems the best way in the future to improve irrigation efficiency in agricultural systems: i.e., determine the irrigation dose from a WB model and then adapt that dosage through the use of sensors to the real situation of each plot. For this purpose, an interactive software-based decision support system (DSS) can be used to help decision-makers compile useful information from a combination of raw data, documents and personal knowledge. This information can then be deployed to identify and solve problems, and make optimized decisions. The simplest DSS designed to carry out automated irrigation consists of activating or deactivating irrigation when the sensor measurements are above or below predefined threshold values [15,20,21,22,23]. A more complex proposal is a DSS which combines the WB method with soil or plant sensors to readjust the ETc [24,25,26,27]. Millán et al. [28] used a DSS that executed a pre-established irrigation scheduling in which RDI was applied without human intervention in a plum crop.

One aspect that complicates the efficient irrigation management of crops is plot heterogeneity, which depends on factors such as plot size, soil characteristics, orography, previous plot uses, etc. If the problem of soil spatial variability is not taken into account, an irrigation design may not be efficient [29]. Work on experimental plots rarely addresses these problems, which require the use of specialist tools to characterize spatial variability. Remote sensing and soil mapping are tools that can be used for agronomic crop management, allowing characterization of the development of the vegetation cover and the large-scale water status of the crop. Very interesting results have been obtained for olive groves with such tools [30]. Using data related to the electrical properties of the soil and multispectral images, easily available at high resolution, can be the best option to delineate different homogeneous zones. For instance, Pedrera-Parrilla et al. [31] and Moral et al. [32] used soil electrical data for zoning purposes, and Hall and Wilson [33] and Martínez-Casanovas et al. [34] utilized vegetation indices computed from multispectral images. The use of satellite data to evaluate changes in vegetation properties aimed at applications in precision agriculture has also been investigated [35,36]. Coarse resolution satellite images are useful tools to describe the phenology of the vegetation [37]. Crop monitoring offers direct information for the analysis of the spatial variability of the crop area. On the basis of such information management actions can be taken to improve production practices. The information can be used to adjust and direct fertilization, determine crop development, adapt irrigation to the needs of the crop, and schedule the harvest. One way to determine crop development is based on the use of reflectance measurements, which differ depending on the type of surface and, in the case of plants, the species, cultivar, and plant status [38]. From this information, the normalized difference vegetation index (NDVI) can be computed (ratio of the difference of the values of reflectance in the near infrared and red bands and their sum). It is known that NDVI is a good indicator of the state of development of vegetation and, in consequence, can be used to detect canopy differences within a field [39].

The objectives of this study were to test the technical feasibility and to evaluate the productive response of a heterogeneous plot in an SHD olive grove cv. ‘*Arbequina’* when using a DSS to carry out a fully automated irrigation scheduling and implementing RDI strategies based on the information obtained through remote sensing and soil monitoring.

## 2. Materials and Methods

### 2.1. Site Description and Experimental Design

This work was performed over the course of three years (2015–2017) in a 9.31 ha commercial hedgerow olive grove (*Olea europaea* L.) planted with cv. *‘Arbequina’*. The farm is located in the municipality of Alvarado, about 16 km east of Badajoz in southwestern Spain (38°49′27.15″ N, 6°46′ 18.39″ W, datum WGS84). The trees were planted in autumn 2007 at a density of 1.852 olive trees/ha (4 × 1.35 m), with a north-south orientation and trained to a central axis. The predominant soil in this field was classified as a Haplic Fluvisol according to the FAO (2006). Soil maintenance involved the practice of non-tillage and the application of herbicides to ensure it remained free of weeds. The climate of the area is Mediterranean with a mild Atlantic influence, with a dry season from June to September (summer) and a wet season from October to May (winter) in which 80% of total precipitation falls. Average ETo and precipitation *p*-values in the area were 1188.25 mm and 503.56 mm, respectively, for the 2007–2017 period. For the same period, the average maximum and minimum air temperature values were 23.49 °C and 9.56 °C, respectively. The hottest months are July and August. Maximum temperatures of over 40 °C are recorded nearly every year, with peak values rarely over 45 °C. The coolest months are December and January. Temperatures below 0 °C are recorded every year, with minimum values rarely below −5 °C. The meteorological information was obtained from a weather station located in the same study plot at a height of 3 m above ground level (in line with a tree row).

Trees were irrigated daily using a drip system with a single lateral line per tree row located close to the base of the tree, with pressure-compensating drippers spaced at 0.67 m and with 1.6 L h^−^¹ discharge rates. The plot is described in more detail in Millán et al. [40].

### 2.2. Characterization of Spatial Variability of the Plot, Selection of Control Points and Soil Analysis

One very important aspect, which complicates the efficient irrigation management of crops, is plot heterogeneity. When establishing an automatic irrigation system it is, therefore, important to know the spatial variation in soil and crop development. A satellite image and a Dualem-1S non-contact sensor (Dualem, Inc., Milton, ON, Canada) were used to evaluate the heterogeneity of the plot. The NDVI measurement was provided by the Sentinel-2A (European Space Agency, ESA) satellite with an image taken in August of 2015 with no cloud cover. The image was processed and analyzed in QGIS 2.18 (https://www.qgis.org) using DOS1 atmospheric correction.

The Dualem-1S sensor was used to measure apparent electrical conductivity (ECa) and was equipped with a global positioning system (GPS) antenna. The sensor comprised a transmitter operating at a frequency of 9 kHz and two receivers with different orientations. There was a 1 m separation between the transmitter (Tx) and the two receivers (Rx). The ECa was measured at 0–0.50 m and 0–1.50 m depths. The sensor was introduced into a 3 m long polyvinyl chloride (PVC) structure which was transported by a pickup truck traveling at an average speed of 9 km h^−^¹. Due to the height of the vehicle, ECa readings were carried out of the 0–0.40 m and 0–1.40 m soil layers, assuming that the ECa of the air is zero. In this study, we used the ECa measurements of the top layer (0–0.40 m). The ECa measurements were taken in August 2015 along different parallel transects that were approximately 4 m apart. The Dualem-1S was programmed to register measurements each second, and a total of 3313 ECa measurements were obtained in the plot. Ordinary kriging was used to develop the ECa map.

**Figure 1:**
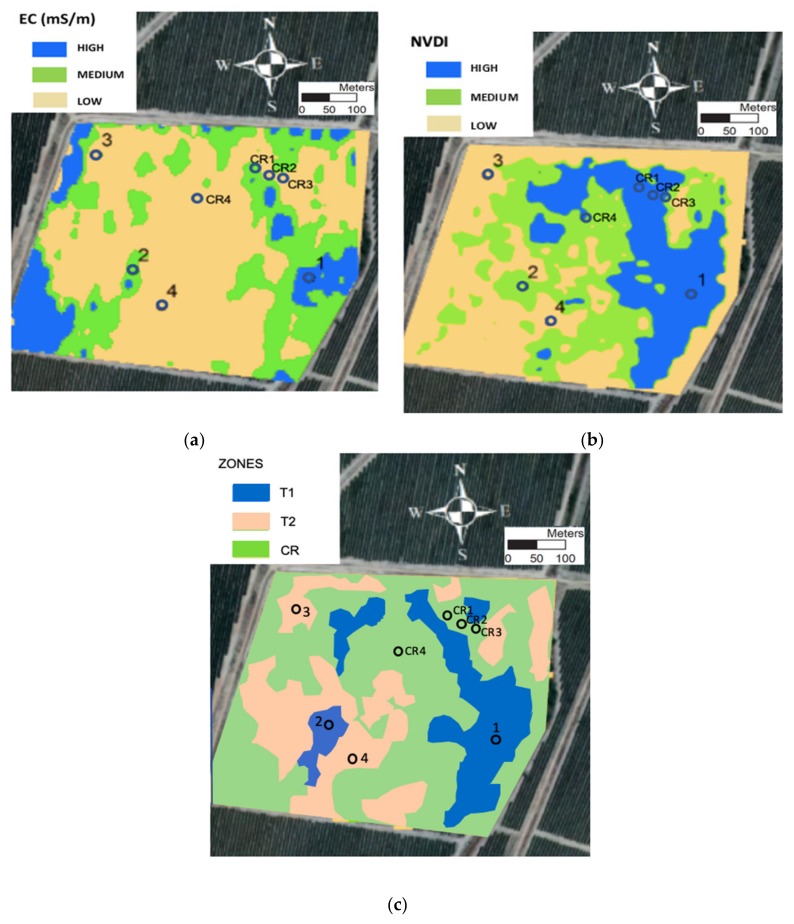
(**a**) Map of soil apparent electrical conductivity (EC) measured with a Dualem-1S sensor. High EC values are between 15–20 (mS/m), medium values between 10–15 (mS/m) and low values between 5–10 (mS/m); (**b**) Normalized difference vegetation index (NDVI) map using a satellite image obtained from Sentinel-2A. High NDVI values are between 0.45–0.50, medium values between 0.40–0.45 and low values between 0.35–0.40, and; (**c**) maps of the different zones established in the study area. Circles 1, 2, 3 and 4 indicate the locations of the sampling sites where irrigation was managed manually, and circles CR1, CR2, CR3 and CR4 sites where irrigation was applied automatically.

Figure 1a shows the soil ECa map and Figure 1b the NDVI map. ECa is related to different soil characteristics that directly or indirectly influence the availability of water for cultivation. The map was made in August when the water content was mostly from irrigation. These served as the basis for determining the control points in the study plot. As seen in Figure 1a, the values classified as low, medium and high (because the ECa values may change with different water contents in the plot) allowed identification of areas with different ECa values in the plot. The highest ECa values were found in the southwestern, northwestern and eastern areas of the experimental field, corresponding to a higher water retention capacity. Soil texture was one of variables considered in the geostatistical analysis for identifying the management zones in the plot [40]. Analysis of the satellite image (Figure 1b) allowed identification of the areas with highest and lowest canopy crop development. The NDVI showed the highest values to be in the northern and central areas of the field. These intra-field ECa and vigor differences are due to a complex interaction of biological, agronomic, edaphic, anthropogenic, topographic and climatic factors [32].

With the information obtained from Figure 1a,b, a new map was generated (Figure 1c) using the map algebra tool (raster calculator) in QGIS 2.18. As can be seen in Figure 1c, three different zones were established:

- Zone 1 (T1): where the ECa and NDVI values were medium or high. The sampling points 1 and 2 were found in this zone.
- Zone 2 (T2): where the ECa and NDVI values were low. The sampling points 3 and 4 were found in this zone.
- Zone 3 (CR): where the ECa values were low and the NDVI values were medium or high. The sampling control points CR1, CR2, CR3 and CR4 were found in this zone.

The maps made during the first year served as a basis to establish in the following seasons in situ control areas. Each control area consisted of 3 rows of 6 trees each. The various measurements that were subsequently taken were made in the 4 central trees of each control area.

The DSS-managed automatic irrigation system was established in Zone 3. This had the most unfavorable conditions for the hedgerow olive grove and corresponded to places with medium or high vigor and low ECa values. In this zone, three control points were selected CR1, CR2, CR3 (2016 and 2017) (Figure 1) and in 2017 one more control point, CR4 (Figure 1), was added to check the system in different soil and crop conditions

Zones 1 and 2 were used to compare the irrigation scheduling carried out in Zone 3. These two first zones were irrigated according to the expert technical criteria. In both zones, two sampling points were selected, in Zone 1 (T1) points 1 and 2, and in Zone 2 (T2) points 3 and 4 (Figure 1). At these points, irrigation volumes were recorded daily using digital water meters (CZ2000-3M, Contazara, Zaragoza, Spain).

Table 1 shows the soil properties in the study area. Soil samples were collected on 15 November 2015 at the different control points whose coordinates were determined using a Mesa-Geode positioning system (Juniper Systems, Logan, UT, USA) at two depths: at 0.0–0.30 m and at 0.30–0.60 m. All soil samples were transported to the lab in plastic bags and were air-dried, ground, and passed through a 2 mm sieve. The soil was characterized in terms of texture, pH and organic matter (OM). Soil texture was determined by mechanical analysis with the hydrometer method [41], pH was measured in a 1:2.5 (soil: water) suspension using the potentiometric method [42] and OM was determined by dichromate oxidation [43].

### 2.3. Decision Support System (DSS)

In order to carry out the automatic irrigation, the DSS comprised two components: (a) sensors installed in the field and (b) IRRIX, a web platform whose algorithm is based on a combination of the water balance with soil moisture sensor feedback adjustment mechanisms.

**Figure 2:**
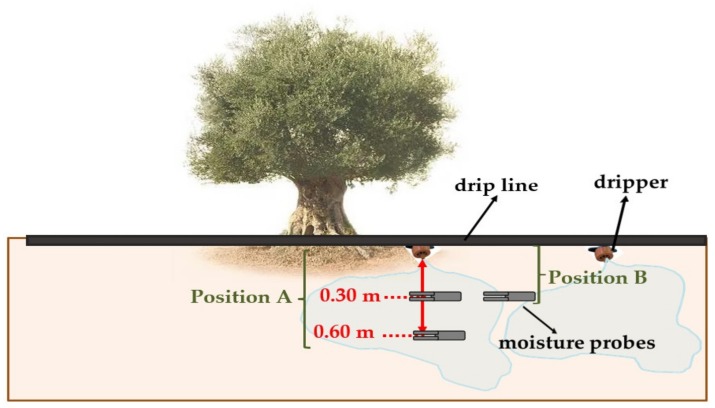
Sensor location.

(a) Sensors installed in the field: to monitor the soil moisture, 10 HS capacitive moisture sensors (Decagon Devices Inc., Pullman, WA, USA) were installed at different positions (position A and position B) (Figure 2) in the different control points selected (CR1, CR2, CR3 and CR4). Two drippers were monitored at each control point. Four moisture sensors were placed under each dripper in the position A, two at a depth of 0.30 m and the others at a depth of 0.60 m. In addition, one measure sensor was situated between the two drippers in the position B at a depth of 0.30 m (Figure 2). These 5 moisture sensors were installed in each of the control points (CR1, CR2 and CR3) in 2016, making a total of 15 sensors. In 2017, the number of soil moisture sensors was increased from 15 to 20, as a new control point was added (CR4). When an error was detected in any of the sensors that had been installed, that sensor was automatically replaced with another in the same position.

A solenoid valve (Rain Bird Europe SCN, Aix-en-Provence, France) and pulse water meter (Lab-Ferrer S.L., Cervera, Lleida, Spain) were also installed at each control point to measure and monitor each irrigation event. An air temperature sensor (CS2015, Campbell Scientific Inc., Logan, UT, USA) was also installed in a central point of the zone. All sensors were connected to a datalogger (CR1000, Campbell Scientific Inc., Logan, UT, USA) via cables. In addition, a voltage regulator (BlueSolar PWM-Pro, Vitron Energy BluePower, The Netherlands) and a relay module (SMD-CD16AC, Campbell Scientific Inc., Logan, UT, USA) were also connected to the datalogger to control the opening and closing of the solenoid valves according to the programming established by the system. The program used in the datalogger was written in CR Basic (Campbell Scientific Inc., Logan, UT, USA) and implemented the functionalities of an irrigation automata. All data were stored each 5 min. The data was downloaded to an IRRIX server four times a day. 

The distance between the different control points for automatic irrigation was limited by the maximum possible cable distance to maintain the electrical signal with sufficient quality. With this limitation, it was decided to locate the control points in the same area, the most disadvantaged one, which required more precise control of the irrigation (sandier texture and more vigorous trees).

**Figure 3:**
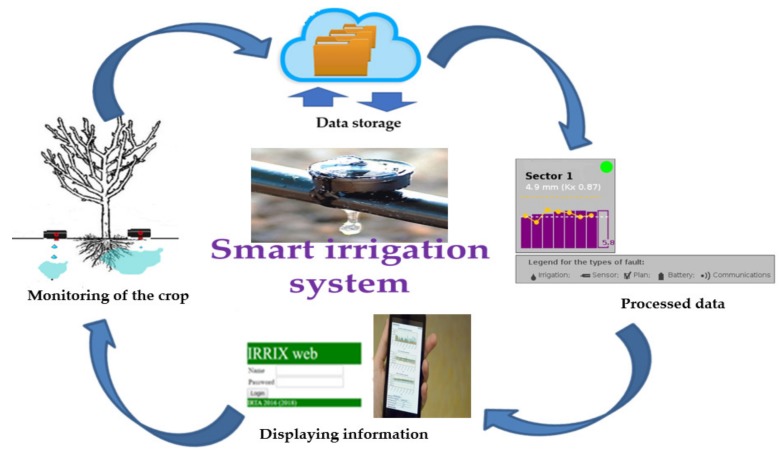
Tasks carried out by IRRIX.

(b) IRRIX is a cloud-hosted web platform that carries out the following daily tasks:

1. Data collection of sensors installed in the field (Figure 3). IRRIX downloads sensor data at periodic intervals throughout the day and at the user’s request.
2. Analysis of all data and calculation of irrigation water volumes. Once a day, IRRIX analyses the set of data to determine the irrigation dose using the information provided by the moisture sensors. To achieve this, this tool integrates an algorithm which combines a WB-based estimation of crop water needs (feed-forward control) with readjustment based on sensor readings (feedback control). [25,28,44]
3. Irrigation scheduling. IRRIX sends the updated irrigation doses to the datalogger. Then, this device orders the activation of the rest of the equipment (solenoid valve or pumps, etc.) to apply the required irrigation doses.
4. Interaction with users. IRRIX is an autonomous system whose main objective is to free the user from work. The main function of the user is to check that the system has worked correctly. Logically, if there is any anomaly in the system it has to be resolved by the user.

Before starting the irrigation campaign, the user must input to IRRIX a plan that provides a rough estimation of how the water will be distributed throughout the campaign. This seasonal plan is drawn up by an expert who determines the guidelines that the irrigation must follow, in this case taking into account the deficit periods. This plan represents the cumulative irrigation water amount throughout the campaign. In order to adapt to the conditions of each year, upper and lower limits are set to this baseline. For this, a set of curves has to be defined, with the automated control system positioned between those curves in such a way that the system must be between a maximum and a minimum cumulative irrigation value.

### 2.4. Irrigation Scheduling

The irrigation carried out on the plot in the different years was the following:

- In 2015, all the plot zones were irrigated according to the criteria of the farmer.
- In 2016, all the plot zones were irrigated according to expert technical criteria [45]. In the T1 and T2 zones, the irrigation scheduling was under human control (non-automatic irrigation scheduling, NAIS). Irrigation was controlled by solenoid valves operated by a commercial automaton, Agronic 4000 (Sistemes Electrònics Progrés, Palau d’Anglesola, Lleida, Spain), which was programmed remotely, every Monday, using the desktop application provided by the manufacturer. The CR1, CR2 and CR3 control points were irrigated automatically through the IRRIX system (automatic irrigation scheduling, AIS), without human intervention. The scheduled irrigation dose was independent at each control point. The irrigation criterion was the same in both cases: a light RDI to preserve oil yield.

Irrigation scheduling was adjusted according to measurements of midday stem water potential (Ψ_stem_) made in T1 and T2:

○ Phase I, from sprouting (early in March) until the beginning of the olive pit hardening stage (beginning of July) [45]. The threshold established for Phase I was −1.4 MPa [12], which means the implementation of only a slight water deficit.
○ Phase II, from the beginning of the hardening of olive pit until the beginning of veraison (mid-September) [45]. The threshold established for Phase II was −2.0 MPa [12].
○ Phase III, from the end of Phase II until harvest [45]. The threshold established for Phase III was −1.6 MPa [12].

For T1 and T2, the ETo was calculated according to the formula of Penman and Monteith [14], modified using the data obtained through an external meteorological network (REDAREX, http://redarexplus.gobex.es), and weekly Ψ_stem_ measurements were used to adjust irrigation.

**Figure 4:**
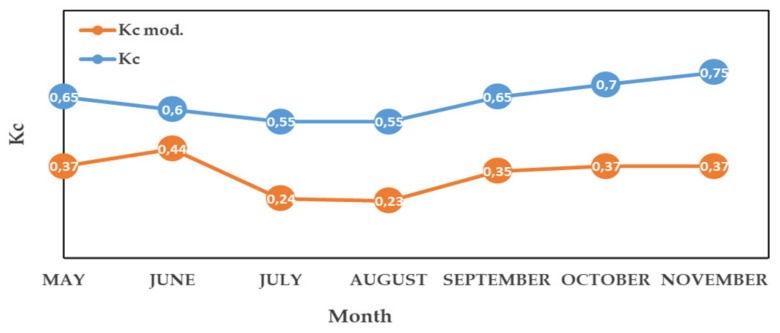
Crop coefficient (Kc) values according to the physiological state of the olive grove. Kc was adjusted to the crop conditions of Extremadura and was calculated with the Orgaz et al. [47] method using the 10-year average, and Kc mod. was adapted to the deficit irrigation strategy.

In the case of CR1, CR2 and CR3, the ETo was estimated daily from the air temperature sensor using the Hargreaves equation [46] and Ψ_stem_ values were not used to adjust irrigation. To carry out irrigation at these points through the DSS, the olive irrigation expert [45] provided a modified Kc (Kc mod.) that took into account the deficit irrigation strategy. This Kc mod., which has to be introduced into IRRIX at the beginning of the irrigation campaign (Figure 4) for the preparation of the seasonal plan, was established on the basis of technical experience in the same plot with a view to attaining the target Ψ_stem_ for each of the previously described phases. At the CR1, CR2 and CR3 points, irrigation scheduling was carried out independently and aimed to simulate the irrigation that was carried out in T1 and T2.

- In 2017, irrigation scheduling was similar to that of the previous year, but one more control point was added (CR4) where automatic irrigation was also carried out. As in the other CR points, the CR4 irrigation scheduling was carried out independently. The CR1 and CR2 automatic irrigation scheduling was carried out in the DSS on the basis of the information provided by the sensors located in CR2. In accordance with the evolution of Ψ_stem_, a series of adjustments were made in 2017 in relation to the seasonal plan (due to the fact that this year was unusually dry) and the soil comfort zone in relation to the sensor readings. This soil comfort zone specifies to the control system the acceptable range for the soil moisture sensor measurements and their pre-established boundaries were empirically readjusted to fit with the observed range.

A summary of the irrigation scheduling followed in this study is shown in Table 2.

### 2.5. Physiological and Agronomic Measurements

#### 2.5.1. Water Status and Canopy Volume

The Ψ_stem_ was measured once a week with a pressure chamber (Model 3005, Soil Moisture Equipment Corp., Santa Barbara, CA, USA). One shaded-leaf per tree was selected near the base of the trunk and covered with aluminum foil at least two hours before the measurements [48]. This selection was carried out between 12:00 and 13:00 h solar time. Determinations were made in four trees per control point. The average Ψ_stem_ value was used to calculate the irrigation correction in T1 and T2. This average was compared with the threshold value and the deviation was calculated.

#### 2.5.2. Yield Data and Oil Content

In 2015, yield measurements were made for the T1, T2, CR1, CR2 and CR3 sampling points. The yield control points were increased by one (CR4) in 2017. At each control point, 4 olive trees were hand harvested when the trees reached a maturity index (MI) of approximately 2.5. A weekly sampling was carried out in every control point from the beginning of veraison. On each occasion, a sample of one hundred olives was classified in eight color groups, and the MI was calculated according to the procedure described in [49]. The yield of each tree was weighed separately, and subsamples of 100 olive fruits from the total harvest of the four trees were collected to determine the average weight of the fruit and the number of fruits per tree. All subsamples were weighed fresh. Oil content was measured for a 1 kg sample of the four trees by Soxhlet extraction in accordance with EEC Regulation 2568/1991 [50]. For this, the sample was crushed and dried in a DryBig 250 oven (Borel Fours Industriels and Etuves, S.A., Neuchâtel, Switzerland) at 105 °C. In this study, the trees were harvested in late October and early November.

### 2.6. Statistical Analysis

An analysis of variance (ANOVA) was used for the statistical analysis of the data. When significant differences were detected, a comparison of means was made applying Duncan’s test at *p* < 0.05. The statistical package IBM SPSS version 24.0 for Windows (IBM Corp. Armonk, NY, USA) was used.

## 3. Results and Discussion

### 3.1. Climatic Conditions

Table 3 shows the mean temperature (T_mean_) and mean relative humidity of the air (RH_mean_) for each of the three phases in the three study years as well as the annual mean values, and the cumulative rainfall, ETc and ETo (calculated using the Penman–Monteith (ETo-PM) and Hargreaves (ETo-H) methods) values for each of the three phases in the three study years as well as the annual cumulative values. The highest Tmean and lowest RH_mean_ values were observed in 2017. The highest T_mean_ values were reached in Phase II (summer months) in all years, and the lowest in Phase III in 2015 and 2017 and in Phase I in 2016. Cumulative rainfall in the experimental period amounted to 181 mm in 2015, 152 mm in 2016 and 76 mm in 2017. Annual cumulative rainfall ranged from 475 mm (2016), the rainiest year, to 265 mm (2017), the driest year as well as the year with the highest annual ETo values. The annual ETo-PM was very similar in the three years of study, with higher values in Phase I. The ETo-H followed the same seasonal trend as ETo-PM, although with slightly higher values. The ETo-H overestimated the ETo-PM by 7% on average. The annual ETc was 834 mm, 861 mm and 894 mm for 2015, 2016 and 2017, respectively.

### 3.2. Applied Water

Table 4 shows the irrigation amounts supplied in each phase in the three study years. It should be noted that 2017 was unusually dry, and so the amount of applied irrigation water was higher due to the low rainfall and high ETo. Comparing the different control points in each zone, it is possible to observe how in 2015 the irrigation applied was very similar in all cases for each Phase and in total. In 2016, the AIS zones received less water than T1 and T2, mainly in Phase II with 16% less, with a consequent total water saving of 24%. It should be noted that at sampling point 4 (in T2) there was an irrigation system failure during Phase II and, therefore, the water applied in that phase was lower than for points 1, 2 and 3 in T1 and T2. In 2017, AIS applied more water in Phase I in CR1, CR2 and CR3 points and less water in Phases II and III than the amounts applied at the T1, T2, T3 and T4 points. It is observed that the AIS emulates the expert scheduling and obtained a 6% reduction in applied water compared to the NAIS system. The DSS schedules irrigation independently at each of the points in accordance with the particular conditions that exist in each zone.

The ability of the automatic system to respond differently in each of the control points may, therefore, respond to differences in soil water reserves which depend on soil texture and crop water demand at each particular point. This information is provided continuously by the frequency domain reflectometry (FDR) sensors. However, the greater or lesser presence of roots in the area where the sensors are located also needs to be taken into account. In addition, it is important to consider the size of the roots as this can affect the readings of the FDR sensors, with an increase in root system size resulting in increasingly larger underestimations of the true volumetric water content, presumably because of the water in the roots [51]. With regard to the (rain + irrigation)/crop evapotranspiration relationship (Table 4), the crop received less water in relation to its needs in 2015, when irrigation was carried out at the farmer’s discretion.

**Figure 5:**
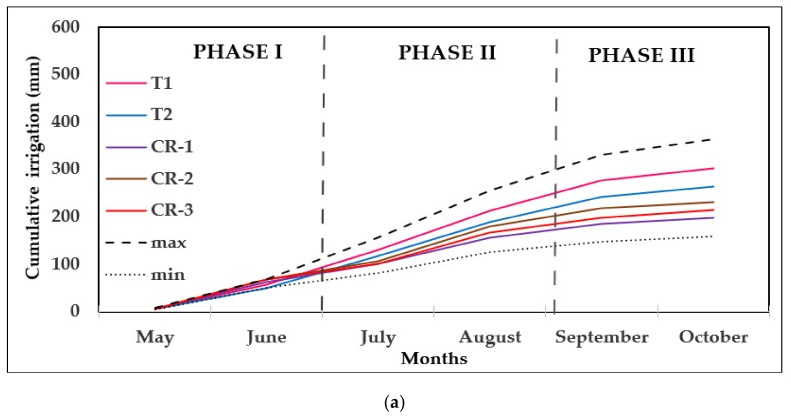

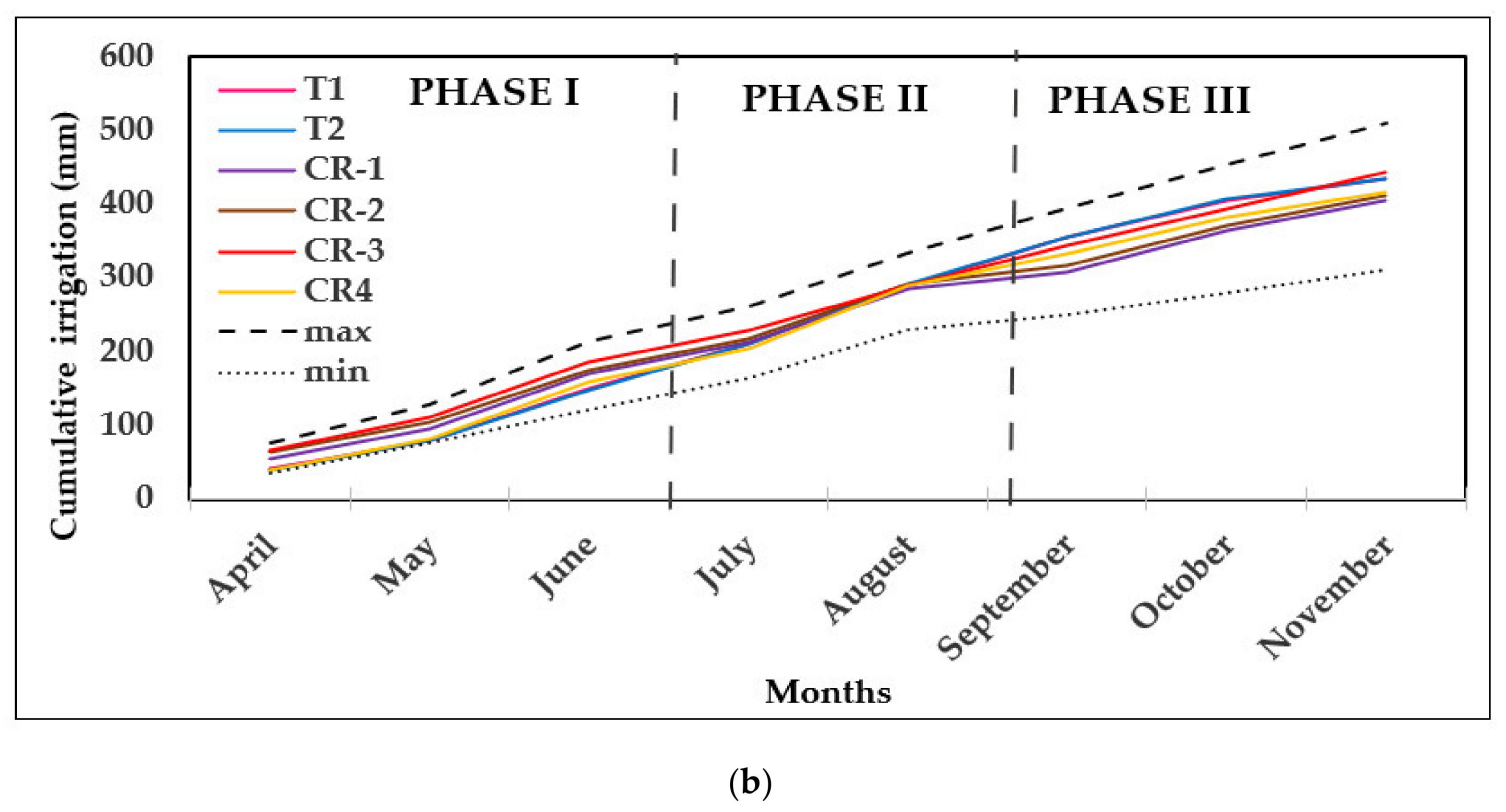
Cumulative irrigation in the different zones (**a**) 2016 and (**b**) 2017. max and min correspond respectively to the maximum and minimum limits set in the seasonal plan.

Figure 5 shows cumulative irrigation at the NASI and AIS points in 2016 and 2017. The irrigation amount was lower in 2016 than in 2017, as 2017 was a drier year. In 2016, the water applied until early July was very similar in all points but, after this date, more water began to be applied at T1 and less water at CR1. In 2017, the irrigation season started a month earlier and the initial irrigations differed at the different points until converging in August. From that date onwards, more water was applied at CR3 than the rest of the points until the end of the irrigation campaign. As water volumes were higher in 2017, the maximum and minimum limits are also higher as they were established as percentages of the campaign schedule. The irrigation pattern was different at each CR point, with the greatest differences observed in Phase II in the case of CR1, CR2 and CR3. In the case of CR4, the pattern was different from the rest: more water was applied in Phase II and less in Phase III. In summary, the water applied generally followed the same pattern in the different control points in 2016, while in 2017 there was a change in trend during Phase II.

### 3.3. Soil Water Content

With respect to soil water content, Table 5 specifies the high and low reference values of each of the moisture sensors during the years 2016 and 2017. IRRIX establishes two parameters for each sensor: a high reference (field capacity, FC) and a low reference (in deficit irrigation conditions). The high reference represents the value recorded by that sensor under soil FC conditions, and this value can be obtained out of season after rain. The low reference is determined for each sensor at the driest value recorded every day. A more detailed description can be found in Millán et al. [28] of how IRRIX determines the high and low reference for each sensor. These high and low reference values serve to reduce the variability between the sensors and to obtain homogeneous reference points for the different sensors. It can be seen in Table 6 that the high reference value of each sensor was higher in 2016 than in 2017, with the exception of sensors 1 (S1) and 3 (S3) located in CR1 and sensor 10 (S10) in CR2. As for the low reference, the values are lower in 2016 than in 2017 except for the sensors that are located in position A at 0.60 m in CR1 and CR2 and sensor 12 (S12) in CR3. The references established for each sensor changed every year, as IRRIX is a dynamic system that adapts to the conditions of each campaign and even to changes during the campaign itself.

**Figure 6:**
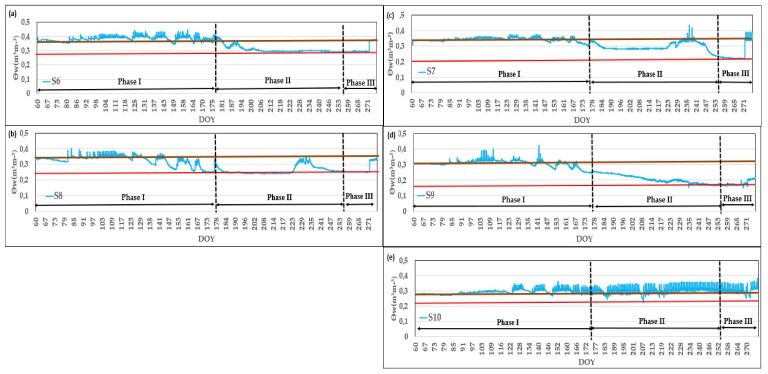
Temporal trends of soil moisture measured by moisture sensors located in different positions: (**a**) A at 0.3 m depth, (**b**) A at 0.3 m depth, (**c**) A at 0.6 m depth, (**d**) A at 0.6 m depth and (**e**) B at 0.3 m depth. Θw is the moisture content. All the probes were installed at CR2 for the automatic irrigation system. The brown line corresponds to the high reference value of the sensor and the red line to the low reference value. The period represented corresponds to the irrigation campaign in the year 2017. DOY is the day of the year.

The temporal evolution of soil water content (SWC) is presented in Figure 6 for control point CR2 in 2017. With respect to the position under dripper (A) at 0.3 m depth (Figure 6a,b), the sensors followed the same overall pattern. These sensors are very sensitive to irrigation, responding quickly even to low volumes of rain or irrigation. Furthermore, these sensors had a very marked response amplitude between minimums before irrigation and maximums at the end of irrigation. It can be seen in Figure 6b that, in Phase II, sensor 8 (S8) presented a different behavior to sensor 6 (S6) (Figure 6a), as the S8 responded to applied irrigation during a certain period of time. This result may be attributable to the place where the emitter was located because of the spatial variability of the moisture distribution in the soil.

Probes located at 0.6 m depth (Figure 6c,d) showed less difference between the maximum and minimum and also responded quickly to small amounts of rain as did the probes located at 0.3 m. The two sensors (S7 and S9) showed different behavior throughout the study period. Normally, sensors installed in the same position follow the same pattern but, as sometimes these sensors show a totally different behavior, consideration needs to be given to sensor-to-sensor variability. This variability is greater in the case of localized irrigation, since wet bulbs are formed around the emitters while the rest of the soil may be only slightly affected or not at all by the irrigation. Due to the existence of spatial variability, sensor variability can be aggravated in the case of woody crops since the moisture sensors need to be installed in the area of the bulb where there may be more roots [25,28]. Kang et al. [51] indicated that the size of the root can affect the readings of FDR sensors. A careful interpretation is therefore required when FDR sensors are used to determine soil moisture content. Sensor variability can also be due to changes that occur in the dielectric properties of the soil [52] or other soil properties, especially bulk density and the existence of macropores.

The sensors installed in position A at 0.3 m depth presented a range of very different measurements. For example, the S6 had maximum values of 0.447 m^3^/m^3^ with a measurement range (the measurement range is the sensor reading at the beginning and end of the irrigation season) of about 0.371 to 0.389 m^3^/m^3^ while the S8 presented maximum values of 0.385 m^3^/m^3^ with a measurement range of 0.331 to 0.334 m^3^/m^3^. At 0.6 m depth, the measurement ranges between sensors located under the same position also varied considerably. The S7 had maximum values of 0.419 m^3^/m^3^ with a measurement range of about 0.309 to 0.338 m^3^/m^3^ and the S9 presented maximum values of 0.394 m^3^/m^3^ with a measurement range of 0.213 to 0.307 m^3^/m^3^. It must be considered that as sensor depth increases the presence of water in the lower layers is greater, which may be due to the arrival of water from other points. In addition, soil moisture fluctuations decreased with depth. Mittelbach et al. [53] established that 10HS sensors do not capture the daily soil moisture content fluctuations for moist conditions and that soil moisture fluctuations are almost negligible at 0.25 m depth and below.

In the position between drippers (B) at 0.30 m depth (Figure 6e), the sensors responded to low applications of rain or irrigation. It can be seen how sensor 10 (S10) was sensitive to small irrigation amounts and showed a greater amplitude between the maximum and minimum during the study period. In the case of S10, the values of the measurements of the 10HS probes did not surpass 0.371 m^3^/m^3^. This may mainly be due to the creation of preferential channels in the soil and, because of this, the water from one or more drippers moving to the place where the sensor is located.

It should be noted that Phase I corresponds to a period of mild stress, and a slight decrease in the amount of water in the soil is observed in Figure 6a,c,d and a more pronounced decrease in Figure 6b. In Phase II there is a clear decrease in SWC in Figure 6a–d. In Phase III there is a delay in the response to the increase in irrigation that coincides with the onset of rainfall.

### 3.4. Crop Water Status and Productivity

**Figure 7:**
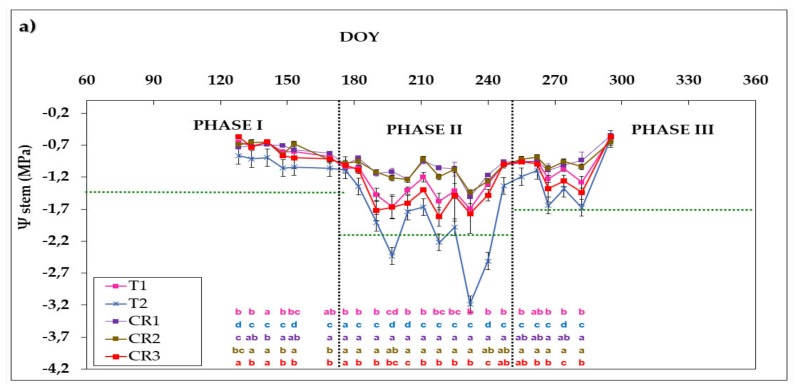

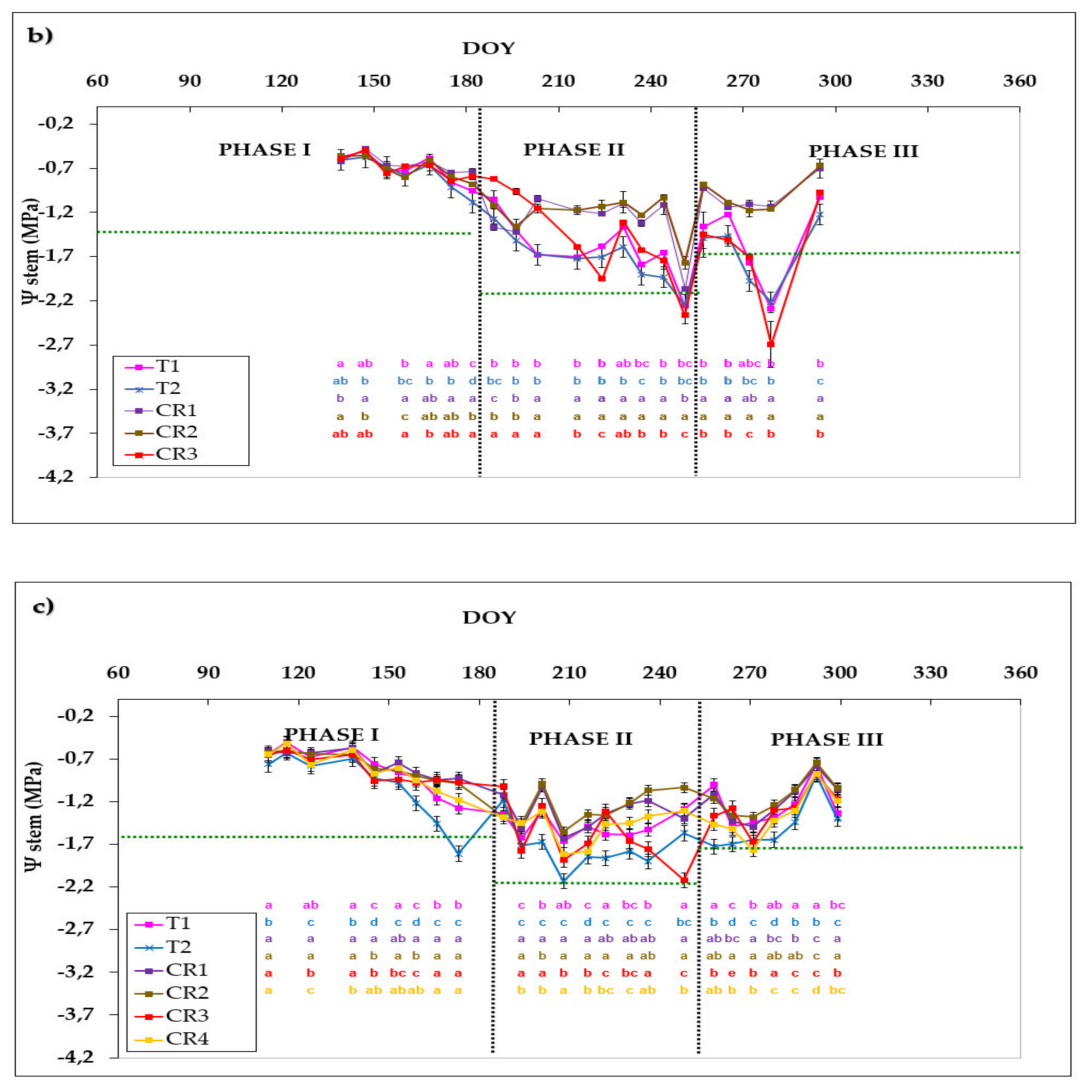
Seasonal patterns of midday stem water potential (Ψ_stem_) during the 2015 (**a**), 2016 (**b**) and 2017 (**c**) seasons in representative trees at each control point. Each value is the mean of four measurements ± standard error. The vertical dashed line indicates the moment at which each phase ends. The horizontal dashed line indicates the threshold established for each crop phase. Different letters in the same column indicate statistically significant differences according to Duncan’s multiple range test (*p* < 0.05). Letters are not shown when no differences were found. DOY is the day of the year.

Figure 7 shows the Ψ_stem_ evolution in 2015 (Figure 7a), 2016 (Figure 7b) and 2017 (Figure 7c) for all control points. A declining trend of Ψ_stem_ can be seen in Phases I and II as the irrigation season advanced, followed by an increase in Phase III to the end of the irrigation campaign. In 2015 (Figure 7a), when all the areas were irrigated according to the farmer’s criteria, there was a marked difference in Ψ_stem_ at the different control points. At all points, Ψ_stem_ was above the reference threshold value in all three phases (shown by a horizontal dotted line in all the figures), except in zone T2 which fell below it on several days in Phase II. The highest values, recorded in Phase I at the beginning of spring, were around −0.66 MPa, −0.87 MPa, −0.79 MPa, −0.70 MPa and −0.57 MPa for T1, T2, CR1, CR2 and CR3, respectively. The minimum Ψ_stem_ was at mid-summer in Phase II with average values of −1.67, −2.43 MPa, −1.12 MPa, −1.22 MPa and −1.67 MPa in T1, T2, CR1, CR2 and CR3, respectively.

In 2016 (Figure 7b), when introducing the expert criteria for irrigation scheduling, the differences in water status between the different control points throughout the entire irrigation season decreased, but especially in Phase II. Higher Ψ_stem_ values were obtained in CR1 and CR2 than the other control points throughout the irrigation season, while CR3 presented the lowest Ψ_stem_ values of around −2.36 and −2.69 MPa reaching the pre-established threshold in Phase II and Phase III, respectively. T1 and T2 also remained above the pre-established limits throughout the irrigation season, except for the days on which CR3 remained below the pre-established limits in Phase II and Phase III, because there was an irrigation failure.

In 2017, the AIS achieved a greater adjustment at CR1 and CR2 to the Ψ_stem_ targets. CR4 had a similar evolution of Ψ_stem_ to the other control points. Greater homogeneity in crop water status between the different points was achieved in 2017 than in 2016.

The Ψ_stem_ values where AIS was carried out followed the same pattern as the Ψ_stem_ values in T1 and T2 (NAIS) in 2016 and 2017, despite the application of different irrigation volumes, due to dosage readjustment made by the IRRIX system with the information provided by the soil probes. The typical spring rainfall and the accumulation of water reserves in the soil during autumn and winter can make it difficult to induce water stress, and the Ψ_stem_ values can sometimes exceed the proposed thresholds during the irrigation season [13,54,55,56,57,58,59,60]. In addition, the algorithm used in the areas where irrigation was carried out automatically showed a good response to the RDI strategies. While some researchers have previously worked with a web platform whose algorithm results from a combination of the water balance with feedback from soil moisture sensors to perform automatic irrigation [24,25,26], this is the first time that the algorithms used to establish the automated irrigation have been shown to respond well to RDI in an olive grove, maintaining the water status of the plant.

The results for yield, number of fruits per tree, oil yield and water productivity (WP) for olives and for oil are presented in Table 6. In 2015, significant differences between different control points were found in yield, number of fruits per tree, oil yield and WP, with CR2 the least productive and with significant differences with T2. In 2016, CR1 and CR3 showed no significant differences in yield with respect to irrigation carried out with expert supervision. However, CR2 had lower yield, number of fruits per tree, oil yield, WP yield and WP oil yield than the other control points, which could be due to alternation in crop yield between more and less productive years as is common in this cultivar. With regard to 2017, yield increased in all control points, with CR2 the most productive. In addition, the WP yield and the WP oil yield also increased notably compared to the previous two years, with 52 kg of olives and about 8.5 kg of oil produced per cubic meter of water provided in CR2. This can be attributed to the lower crop load level in CR2 in 2015 and 2016. The number of fruits per tree also increased with respect to previous years, being significantly higher in CR1 and CR2. No statistically significant differences were found in oil yield at the different control points.

Introducing the strategy recommended by the expert led to an increase in yield and a tendency to ploy homogenization in 2017, especially with respect to oil production which showed no statistically significant differences between the various control points. Moriana et al. [12] previously demonstrated that the RDI strategy used in this experiment and for this crop presented significant differences in yield or oil yield with respect to a control treatment in which the trees were irrigated with 100% ETc.

The heterogeneity of a commercial plot irrigated with the same criteria causes differences between the various plants on the plot. By characterizing this variability and considering these differences for irrigation scheduling, the use of RDI strategies has been shown to lead to greater plot homogenization. This is of great importance for the use of automated irrigation systems where the selection of the control zone(s) that will govern decision making is important. Automatic irrigation has been shown to be capable of simulating expert judgement in the application of RDI strategies based on crop water status. The system improved its performance after the first year of adjustment. Recent studies have reported that RDI strategies can be advantageous in SHD olive orchards in terms of reducing water applications, decreasing excessive tree vigor, improving irradiance environments and increasing yield, oil quality and water-use efficiency [1,6,61,62,63]. The results of the present study provide evidence that an RDI strategy can be applied through a DSS in a plot of SHD olive orchards with no negative effects on yield, number of fruits per tree, oil yield, WP yield or WP oil yield, if there is prior spatial characterization and pre-established criteria for decision making. Characterization of the spatial variability of the plot is very important in the case of a hedgerow olive grove, since an area with greater vigor is not always related to higher productivity [40]. In addition, a better irrigation management in an area with lower water retention capacity (sandy soil) allows more balanced vegetative growth, which is necessary for hedgerow olive grove productivity [64]. Phase II is considered the most important for an automatic irrigation system, and adjustment of the deficit irrigation dosages determined by a field expert can be better controlled by an automatic system analyzing crop water needs and soil moisture. During this phase, plant water status can be better controlled in more unfavorable zones, enabling all field zones to enjoy the correct water status.

## 4. Conclusions

This work aimed to evaluate and develop an automated irrigation protocol in a hedgerow olive orchard using the IRRIX platform. In this case, the device had to simulate a recommended irrigation strategy for this type of plantation that included the use of regulated deficit irrigation. The characterization of the spatial variability was useful to locate the control points.

The automatic irrigation scheduling simulated the expert criterion, adapting to the specific conditions of the control point where the soil sensors were installed. The total amount of manually and automatically applied irrigation water was similar at the various control points, but not its distribution as the DSS estimated the water needs by combining the water balance method with a feedback mechanism based on the moisture sensor readings. In addition, the DSS was able to establish an RDI strategy which induced moderate-to-severe stress during Phase II of the crop in the parts of the plot with the most unfavorable conditions for the hedgerow (places with high vigor and low ECa). The adoption of an appropriate RDI strategy in an SHD olive grove enabled homogenization of plot yield, with a tendency to increase production. The automatic irrigation allowed irrigation management with minimum human intervention.

The results obtained with the system improved in the third year, when adjustments were made based on the information collected in the previous year. The automated irrigation has proven to be able to adapt to the particular conditions of the place where it is installed and to the different growth stages of the crop, thus improving the key efficiency parameters.

Automatic irrigation scheduling is of particular interest in the case of olive groves. In this crop, monitoring stem water potentials at commercial level for crop phase dosage readjustment is complicated by the strongly negative values that can be attained but which cannot be measured with a pump-up pressure chamber.

Although the results were encouraging, further studies are required to improve certain aspects of the system, particularly relating to the integration of the NDVI and ECa measurements into the DSS.

## Figures and Tables

**Table 1:** Soil analysis in the studied points.

Points	Depth(m)	Sand (%)	Clay (%)	Silt (%)	Texture	OM (%)	pH
1	0.00–0.30	43.91	29.72	26.37	clay-loam	1.18	7.87
	0.30–0.60	64.47	5.25	30.28	sandy loam	0.53	6.27
2	0.00–0.30	72.05	4.43	23.52	sandy loam	1.16	6.83
	0.30–0.60	72.39	4.04	23.57	sandy loam	0.97	6.43
3	0.00–0.30	72.26	2.30	25.44	sandy loam	0.44	7.87
	0.30–0.60	73.65	3.65	22.7	sandy loam	0.43	6.93
4	0.00–0.30	72.88	7.44	19.68	sandy loam	0.51	6.45
	0.30–0.60	71.28	3.62	25.1	sandy loam	0.46	6.14
CR1	0.00–0.30	64.77	12.6	22.63	sandy loam	0.68	7.01
	0.30–0.60	65.81	15.31	18.88	sandy loam	0.55	6.87
CR2	0.00–0.30	64.84	15.81	19.35	sandy loam	0.54	7.15
	0.30–0.60	64.64	15.69	19.67	sandy loam	0.44	6.86
CR3	0.00–0.30	63.66	8.13	28.21	sandy loam	0.94	6.66
	0.30–0.60	61.79	9.02	29.19	sandy loam	0.82	6.65
CR4	0.00–0.30	75.72	6.87	17.41	sandy loam	0.48	5.40
	0.30–0.60	74.25	5.60	20.15	sandy loam	0.46	5.58

OM = organic matter.

**Table 2:** Irrigation scheduling carried out in the different zones.

Year	T1	T2	CR1	CR2	CR3	CR4
2015	Farmer	Farmer	Farmer	Farmer	Farmer	
2016	NAIS	NAIS	AIS	AIS	AIS	
2017	NAIS	NAIS	AIS	AIS	AIS	AIS

NAIS = non-automatic irrigation scheduling; AIS = automatic irrigation scheduling.

**Table 3:** Mean temperature (T_mean_), mean relative humidity (RH_mean_), cumulative rainfall and cumulative evapotranspiration (ETo) and crop evapotranspiration (ETc) for each year and each phase of the crop cycle.

Year	Phases	T_mean_	RH_mean_	Rainfall	ETo-PM	ETo-H	ETc
		(°C)	(%)	(mm)	(mm)	(mm)	(mm)
	Phase I	17.8	63.1	99.1	559.2	598.1	315.8
2015	Phase II	25.1	52.2	12.1	414.5	412.7	238.2
	Phase III	16.3	75.5	141.5	225.2	255.5	188.9
	Annual	16.2	69.5	327.9	1304.7	1391.4	834.3
	Phase I	15.9	70.8	204.2	501.6	519.0	319.8
2016	Phase II	25.8	51.8	10.5	389.7	276.8	241.5
	Phase III	16.7	72.7	121.0	239.1	276.8	197.9
	Annual	16.1	72.1	475.3	1225.1	1340.1	860.6
	Phase I	18.2	62.6	76.4	579.2	597.1	351.1
2017	Phase II	25.8	51.4	25.8	395.1	411.1	267.3
	Phase III	16.7	63.3	51.8	251.2	299.6	189.1
	Annual	16.4	65.9	265.4	1330.5	1433.5	894.2

ETo-PM is the reference evapotranspiration calculated through the equation of Penman–Monteith; ETo-H is the reference evapotranspiration calculated through the equation of Hargreaves; RH is relative humidity; ETc was calculated with the Orgaz et al. [47] method.

**Table 4:** Irrigation supplied at each sampling point in the three study years.

			Irrigation (mm)			(R + I)/ETc
Year	Points	Phase I ¹	Phase II ^2^	Phase III ^3^	TOTAL ⁴	(%)
	1	89	119	44	252	47.08
	2					
2015	3	86	118	44	248	46.60
	4					
	CR1	83	108	41	232	44.68
	CR2	82	108	41	231	44.56
	CR3	81	106	40	227	44.08
	1	64	154	51	300	60.72
	2	69	154	50	306	61.42
2016	3	66	150	47	298	60.49
	4	60	134	43	231	52.71
	CR1	63	113	22	198	48.87
	CR2	68	139	25	232	52.82
	CR3	68	121	26	215	50.85
	1	148	109	174	431	60.85
	2	148	110	177	435	61.30
2017	3	147	111	176	434	61.18
	4	148	110	177	435	61.30
	CR1	169	87	149	405	57.94
	CR2	175	87	148	410	58.50
	CR3	185	85	173	444	62.30
	CR4	113	158	99	414	54.03

¹ From sprouting until beginning of olive pit hardening; ^2^ from the beginning of olive pit hardening until the beginning of veraison; ^3^ from the end of Phase II until harvest; ⁴ total irrigation applied. R is effective rainfall in each year of study; I is total irrigation water applied in the different points; ETc is crop evapotranspiration for the irrigation season and was calculated with the Orgaz et al. [47] method. In 2015, irrigation began on 16 April and ended on 22 October. In 2016, irrigation started on 13 May and ended on 21 October. In 2017, irrigation began on 5 April and ended on 2 November.

**Table 5:** High and low reference values of each of the moisture sensors.

			2016		2017	
Olive Grove	Position	Sensor	High Reference	Low Reference	High Reference	Low Reference
	A at 0.30 m	S1	0.371	0.171	0.399	0.296
	A at 0.60 m	S2	0.362	0.248	0.357	0.221
CR1	A at 0.30 m	S3	0.325	0.150	0.356	0.252
	A at 0.60 m	S4	0.369	0.188	0.318	0.175
	B at 0.30 m	S5	0.314	0.167	0.303	0.246
	A at 0.30 m	S6	0.400	0.280	0.399	0.296
	A at 0.60 m	S7	0.359	0.283	0.357	0.221
CR2	A at 0.30 m	S8	0.385	0.165	0.356	0.252
	A at 0.60 m	S9	0.318	0.238	0.318	0.175
	B at 0.30 m	S10	0.298	0.178	0.303	0.246
	A at 0.30 m	S11	0.337	0.104	0.328	0.183
	A at 0.60 m	S12	0.312	0.202	0.252	0.137
CR3	A at 0.30 m	S13	0.447	0.219	0.365	0.283
	A at 0.60 m	S14	0.313	0.073	0.277	0.133
	B at 0.30 m	S15	0.446	0.227	0.362	0.233
	A at 0.30 m	S16			0.325	0.296
	A at 0.60 m	S17			0.356	0.221
CR4	A at 0.30 m	S18			0.331	0.252
	A at 0.60 m	S19			0.362	0.175
	B at 0.30 m	S20			0.301	0.246

High Reference represents the value recorded by the moisture sensor at conditions of field capacity; Low Reference represents the driest value recorded by the moisture sensor in conditions of deficit irrigation. The high and low reference values of each of the sensors correspond to the beginning of the irrigation campaign in the different years.

**Table 6:** Mean values ± standard error of yield, number of fruits per tree, oil yield and water-use efficiency during the three study years.

	Control Points	2015		2016		2017	
	T1	9105 ± 449	ab	12507 ± 759	a	15575 ± 625	b
	T2	12146 ± 760	a	13284 ± 854	a	17809 ± 725	b
Yield	CR1	10240 ± 2037	ab	9732 ± 1362	a	18244 ± 726	b
(kg/ha)	CR2	6740 ± 1324	b	5490 ± 1274	b	21478 ± 1324	a
	CR3	10788 ± 1453	ab	10937 ± 1687	a	18535 ± 2062	ab
	CR4					15277 ± 683	b
	Significance	*		*		*	
	T1	2160 ± 131	ab	4454 ± 383	a	5208 ± 298	b
	T2	3231 ± 297	a	4580 ± 510	a	6457 ± 230	ab
Number of fruits per tree	CR1	2625 ± 511	ab	2884 ± 302	ab	6714 ± 435	a
	CR2	1628 ± 285	b	1345 ± 388	b	7568 ± 548	a
	CR3	2621 ± 486	ab	3966 ± 1202	a	6449 ± 958	ab
	CR4					5089 ± 164	b
	Significance			*		*	
	T1	1725 ± 84	ab	2211 ± 104	a	3047 ± 61	
	T2	2205 ± 140	a	2423 ± 174	a	3375 ± 171	
	CR1	1362 ± 271	bc	1372 ± 201	b	3090 ± 148	
Oil yield	CR2	1024 ± 201	c	914 ± 230	b	3468 ± 141	
(kg/ha)	CR3	1899 ± 256	ab	2071 ± 276	a	3508 ± 345	
	CR4					3033 ± 154	
	Significance	*		*		n.s.	
	T1	36 ± 1.78	ab	41 ± 2.50	a	36 ± 1.44	c
	T2	49 ± 3.06	a	50 ± 3.22	a	41 ± 1.67	bc
	CR1	44 ± 8.78	ab	49 ± 6.87	a	45 ± 1.79	b
WP yield	CR2	29 ± 5.73	b	23.66 ± 5.49	b	52 ± 3.22	a
(kg/m^3^)	CR3	48 ± 6.40	ab	50.86 ± 7.85	a	42 ± 4.64	bc
	CR4					37 ± 1.65	c
	Significance	*		*		*	
	T1	7 ± 0.33	ab	7.29 ± 0.34	ab	7.03 ± 0.14	b
	T2	8 ± 0.56	a	9.14 ± 0.65	ab	7.76 ± 0.39	ab
	CR1	7 ± 1.43	ab	6.93 ± 1.01	b	7.63 ± 0.37	ab
WP oil yield	CR2	4 ± 0.87	b	3.94 ± 0.99	c	8.45 ± 0.34	a
(kg/m^3^)	CR3	6 ± 1.20	ab	9.63 ± 1.28	a	7.90 ± 0.78	ab
	CR4					7.32 ± 0.37	ab
	Significance	*		*		*	

WP = water productivity. * indicates statistically significant differences according to Duncan’s multiple range test (*p* < 0.05). n.s. = non-significant.

## References

[1] Marra F.P., Marino G., Marchese A., Caruso T. (2016). Effects of different irrigation regimes on a super-high-density olive grove cv. “Arbequina”: Vegetative growth, productivity and polyphenol content of the oil. Irrig. Sci..

[2] Bongi G., Palliotti A.O., Shaffer B., Anderson P.C. (1994). Temperate Crops Volume I. Handbook of Environmental Physiology of Fruit Crops.

[3] Vossen P. (2002). The Potential for Super-High-Density Olive Oil Orchards in California. Olint Mag..

[4] Connor D.J., Fereres E. (2010). The Physiology of Adaptation and Yield Expression in Olive. Hortic. Rev..

[5] Cuevas M.V., Martín-Palomo M.J., Diaz-Espejo A., Torres-Ruiz J.M., Rodriguez-Dominguez C.M., Perez-Martin A., Mejías R.P., Fernández J. (2012). Assessing water stress in a hedgerow olive orchard from sap flow and trunk diameter measurements. Irrig. Sci..

[6] Trentacoste E.R., Calderon F.J., Contreras-Zanessi O., Galarza W., Banco A.P., Puertas C.M. (2019). Effect of Regulated Deficit Irrigation during the Vegetative Growth Period on Shoot Elongation and Oil Yield Components in Olive Hedgerows (*Cv. Arbosana*) Pruned Annually on Alternate Sides in San Juan, Argentina. Irrig. Sci..

[7] Romero R., Muriel J., Garcia I., De La Peña D.M. (2012). Research on automatic irrigation control: State of the art and recent results. Agric. Water Manag..

[8] Singh A. (2014). Conjunctive use of water resources for sustainable irrigated agriculture. J. Hydrol..

[9] Campo M.G.-D. (2011). Summer deficit-irrigation strategies in a hedgerow olive orchard cv. ‘Arbequina’: Effect on fruit characteristics and yield. Irrig. Sci..

[10] Girona J., Mata M., Goldhamer D., Johnson R., DeJong T. (1993). Patterns of Soil and Tree Water Status and Leaf Functioning during Regulated Deficit Irrigation Scheduling in Peach. J. Am. Soc. Hortic. Sci..

[11] Rowland D.L., Faircloth W.H., Payton P., Tissue D., Ferrell J.A., Sorensen R.B., Butts C.L. (2012). Primed acclimation of cultivated peanut (*Arachis hypogaea* L.) through the use of deficit irrigation timed to crop developmental periods. Agric. Water Manag..

[12] Moriana A., Pérez-López D., Prieto M., Ramírez-Santa-Pau M., Rodríguez J.M.P. (2012). Midday stem water potential as a useful tool for estimating irrigation requirements in olive trees. Agric. Water Manag..

[13] Iniesta F., Testi L., Orgaz F., Villalobos F. (2009). The effects of regulated and continuous deficit irrigation on the water use, growth and yield of olive trees. Eur. J. Agron..

[14] Allen R.G., Pereira L.S., Raes D., Smith M. (1998). Crop Evapotranspiration-Guidelines for Computing Crop. Water Requirements-FAO Irrigation and Drainage Paper 56.

[15] Miller L., Vellidis G., Coolong T. (2018). Comparing a Smartphone Irrigation Scheduling Application with Water Balance and Soil Moisture-based Irrigation Methods: Part II—Plasticulture-grown Watermelon. Hort Technol..

[16] Amayreh J., Al-Abed N. (2005). Developing crop coefficients for field-grown tomato (*Lycopersicon esculentum Mill*.) under drip irrigation with black plastic mulch. Agric. Water Manag..

[17] Vienken T., Reboulet E., Leven C., Kreck M., Zschornack L., Dietrich P. (2013). Field comparison of selected methods for vertical soil water content profiling. J. Hydrol..

[18] Dabach S., Shani U., Lazarovitch N. (2015). Optimal tensiometer placement for high-frequency subsurface drip irrigation management in heterogeneous soils. Agric. Water Manag..

[19] Elmaloglou S., Soulis K. (2013). The Effect of Hysteresis on Soil Water Dynamics during Surface Trickle Irrigation in Layered Soils. Glob. Nest J..

[20] Luthra S., Kaledonkar M., Singh O., Tyagi N. (1997). Design and development of an auto irrigation system. Agric. Water Manag..

[21] Miranda F., Yoder R., Wilkerson J., Odhiambo L. (2005). An autonomous controller for site-specific management of fixed irrigation systems. Comput. Electron. Agric..

[22] Cáceres R., Casadesus J., Marfà O. (2007). Adaptation of an Automatic Irrigation-control Tray System for Outdoor Nurseries. Biosyst. Eng..

[23] Boutraa T., Akhkha A., Alshuaibi A., Atta R. (2011). Evaluation of the effectiveness of an automated irrigation system using wheat crops. Agric. Boil. J. North. Am..

[24] Bacci L., Battista P., Rapi B. (2008). An integrated method for irrigation scheduling of potted plants. Sci. Hortic..

[25] Casadesus J., Mata M., Marsal J., Girona J. (2012). A general algorithm for automated scheduling of drip irrigation in tree crops. Comput. Electron. Agric..

[26] Osroosh Y., Peters R.T., Campbell C.S., Zhang Q. (2016). Comparison of irrigation automation algorithms for drip-irrigated apple trees. Comput. Electron. Agric..

[27] Saab M.T.A., Jomaa I., Skaf S., Fahed S., Todorović M. (2019). Assessment of a Smartphone Application for Real-Time Irrigation Scheduling in Mediterranean Environments. Water.

[28] Millán S., Casadesús J., Moñino M.J., Moñino J., Prieto M.H., Moñino M.J., Prieto M.H. (2019). Using Soil Moisture Sensors for Automated Irrigation Scheduling in a Plum Crop. Water.

[29] Fortes R., Millán S., Prieto M.H., Campillo C. (2015). A methodology based on apparent electrical conductivity and guided soil samples to improve irrigation zoning. Precis. Agric..

[30] Berni J., Zarco-Tejada P., Suárez L., González-Dugo V., Fereres E. (2009). Remote Sensing of Vegetation from UAV Platforms using Lightweight Multispectral and Thermal Imaging Sensors. Int. Arch. Photogramm. Remote Sens. Spatial Inform. Sci..

[31] Pedrera-Parrilla A., Martínez G., Espejo-Pérez A.J., Gomez J.A., Giráldez J.V., Vanderlinden K., García-Tejero I.F. (2014). Mapping impaired olive tree development using electromagnetic induction surveys. Plant. Soil.

[32] Moral F.J., Terrón J., Da Silva J.M. (2010). Delineation of management zones using mobile measurements of soil apparent electrical conductivity and multivariate geostatistical techniques. Soil Tillage Res..

[33] Hall A., Wilson M.A. (2012). Object-based analysis of grapevine canopy relationships with winegrape composition and yield in two contrasting vineyards using multitemporal high spatial resolution optical remote sensing. Int. J. Remote Sens..

[34] Martínez-Casasnovas J.A., Agelet-Fernandez J., Arnó J., Ramos M.C. (2012). Analysis of vineyard differential management zones and relation to vine development, grape maturity and quality. Span. J. Agric. Res..

[35] Sibanda M., Mutanga O., Rouget M. (2015). Examining the potential of Sentinel-2 MSI spectral resolution in quantifying above ground biomass across different fertilizer treatments. ISPRS J. Photogramm. Remote Sens..

[36] Thenkabail P.S. (2003). Biophysical and yield information for precision farming from near-real-time and historical Landsat TM images. Int. J. Remote. Sens..

[37] Testa S. (2014). Correcting MODIS 16-day composite NDVI time-series with actual acquisition dates. Eur. J. Remote Sens..

[38] Plant R.E. (2001). Site-specific management: The application of information technology to crop production. Comput. Electron. Agric..

[39] Gomez J.A., Zarco-Tejada P.J., García-Morillo J., Gama J., Soriano M.A. (2011). Determining Biophysical Parameters for Olive Trees Using CASI-Airborne and Quickbird-Satellite Imagery. Agron. J..

[40] Millán S., Moral F.J., Prieto M.H., Pérez-Rodriguez J.M., Campillo C. (2019). Mapping Soil Properties and Delineating Management Zones Based on Electrical Conductivity in a Hedgerow Olive Grove. Trans. ASABE.

[41] Bouyoucos G.J. (1936). Directions for Making Mechanical Analyses of Soils by the Hydrometer Method. Soil Sci..

[42] Egnér H., Riehm H., Domingo W. (1960). Untersuchungen Über Die Chemische Bodenanalyse Als Grundlage Für Die Beurteilung Des Nährstoffzustandes Der Böden. II. Chemische Extraktionsmethoden Zur Phosphor-Und Kaliumbestimmung. Kungliga Lantbrukshögskolans Annaler.

[43] Walkley A., Black I.A. (1934). An Examination of the Degtjareff Method for Determining Soil Organic Matter, and a Proposed Modification of the Chromic Acid Titration Method. Soil Sci..

[44] Niño J.M.D., Oliver-Manera J., Girona J., Casadesús J. (2020). Differential irrigation scheduling by an automated algorithm of water balance tuned by capacitance-type soil moisture sensors. Agric. Water Manag..

[45] Pérez-Rodríguez J., Parras-Cintero J. (2014). Manual Práctico De Riego Del Olivar De Almazara.

[46] Hargreaves G.H., Allen R.G. (2003). History and Evaluation of Hargreaves Evapotranspiration Equation. J. Irrig. Drain. Eng..

[47] Orgaz F., Testi L., Villalobos F., Fereres E. (2005). Water requirements of olive orchards–II: Determination of crop coefficients for irrigation scheduling. Irrig. Sci..

[48] Shackel K.A., Ahmadi H., Biasi W., Buchner R., Goldhamer D., Gurusinghe S., Hasey J., Kester D., Krueger B., Lampinen B. (1997). Plant Water Status as an Index of Irrigation Need in Deciduous Fruit Trees. Hort Technol..

[49] Guzmán E.C., Baeten V., Pierna J.A.F., García-Mesa J.A. (2013). Determination of the olive maturity index of intact fruits using image analysis. J. Food Sci. Technol..

[50] EEC (1991). Characteristics of Olive and Olive Pomace Oils and their Analytical Methods. Regulation EEC/2568/1991. Offic. J. Eur. Commun..

[51] Kang S., Van Iersel M.W., Kim J. (2019). Plant root growth affects FDR soil moisture sensor calibration. Sci. Hortic..

[52] Kizito F., Campbell C., Campbell G., Cobos D., Teare B., Carter B., Hopmans J.W. (2008). Frequency, electrical conductivity and temperature analysis of a low-cost capacitance soil moisture sensor. J. Hydrol..

[53] Mittelbach H., Lehner I., Seneviratne S.I. (2012). Comparison of four soil moisture sensor types under field conditions in Switzerland. J. Hydrol..

[54] Moriana A., Orgaz F., Pastor M., Fereres E. (2003). Yield Responses of a Mature Olive Orchard to Water Deficits. J. Am. Soc. Hortic. Sci..

[55] Grattan S., Berenguer M., Connell J., Polito V., Vossen P. (2006). Olive oil production as influenced by different quantities of applied water. Agric. Water Manag..

[56] Tognetti R., D’Andria R., Lavini A., Morelli G. (2006). The effect of deficit irrigation on crop yield and vegetative development of Olea europaea L. (cvs. Frantoio and Leccino). Eur. J. Agron..

[57] Moriana A., Pérez-López D., Gómez-Rico A., Salvador M.D., Olmedilla N., Ribas F., Fregapane G. (2007). Irrigation scheduling for traditional, low-density olive orchards: Water relations and influence on oil characteristics. Agric. Water Manag..

[58] Fernández J., Green S., Caspari H.W., Diaz-Espejo A., Cuevas M.V. (2007). The use of sap flow measurements for scheduling irrigation in olive, apple and Asian pear trees and in grapevines. Plant. Soil.

[59] Correa-Tedesco G., Rousseaux M.C., Searles P. (2010). Plant growth and yield responses in olive (*Olea europaea*) to different irrigation levels in an arid region of Argentina. Agric. Water Manag..

[60] Campo M.G.-D. (2010). Physiological and Growth Responses to Irrigation of a Newly Established Hedgerow Olive Orchard. Hort Sci..

[61] Fernández J., Perez-Martin A., Torres-Ruiz J.M., Cuevas M.V., Rodriguez-Dominguez C.M., Elsayed-Farag S., Sillero A.M.M., García J., Hernandez-Santana V., Diaz-Espejo A. (2013). A regulated deficit irrigation strategy for hedgerow olive orchards with high plant density. Plant. Soil.

[62] Rosecrance R.C., Krueger W.H., Milliron L., Bloese J., Garcia C., Mori B. (2015). Moderate Regulated Deficit Irrigation can Increase Olive Oil Yields and Decrease Tree Growth in Super High Density ‘Arbequina’ Oolive Orchards. Sci. Hortic..

[63] Hernandez-Santana V., Fernández J., Cuevas M., Perez-Martin A., Diaz-Espejo A. (2017). Photosynthetic limitations by water deficit: Effect on fruit and olive oil yield, leaf area and trunk diameter and its potential use to control vegetative growth of super-high density olive orchards. Agric. Water Manag..

[64] Connor D.J., Campo M.G.-D., Rousseaux M.C., Searles P. (2014). Structure, management and productivity of hedgerow olive orchards: A review. Sci. Hortic..

